# Human Papilloma Virus (HPV) and Fertilization: A Mini Review

**DOI:** 10.3390/medicina54040050

**Published:** 2018-07-27

**Authors:** Konstantinos Zacharis, Christina I. Messini, George Anifandis, George Koukoulis, Maria Satra, Alexandros Daponte

**Affiliations:** 1 School of Health Sciences, Faculty of Medicine, Department of Obstetrics and Gynaecology, University of Thessaly, Biopolis, T.K., 42200 Larissa, Greece; zaxarisk@yahoo.com (K.Z.); pireaschristina@gmail.com (C.I.M.); adaponte@gmail.com (A.D.); 2 School of Health Sciences, Faculty of Medicine, Department of Morphology, University of Thessaly, Biopolis, T.K., 42200 Larissa, Greece; geokoukoulis@gmail.com (G.K.); 3 School of Health Sciences, Faculty of Medicine, Department of Biology, University of Thessaly, Biopolis, T.K., 42200 Larissa, Greece; msatra@med.uth.gr

**Keywords:** HPV, fertility, semen, cryopreservation, assisted reproductive technology, IVF

## Abstract

Human papilloma virus (HPV) is one of the most prevalent viral sexually transmitted diseases. The ability of HPV to induce malignancy in the anogenital tract and stomato-pharyngeal cavity is well documented. Moreover, HPV infection may also affect reproductive health and fertility. Although, the impact of HPV on female fertility has not been thoroughly studied it has been found also to have an impact on semen parameters. Relative information can be obtained from studies investigating the relationship between HPV and pregnancy success. Furthermore, there is an ongoing debate whether HPV alters the efficacy of assisted reproductive technologies. An association between HPV and assisted reproductive technologies (ART) programs has been reported. Nevertheless, due to conflicting data and the small number of existing studies further research is required. It remains to be clarified whether HPV detection and genotyping could be included in the diagnostic procedures in couples undergoing in vitro fertilization (IVF)/intrauterine insemination (IUI) treatments. Vaccination of both genders against HPV can reduce the prevalence of HPV infection and eliminate its implications on human fertility. The aim of the present mini-review is to reiterate the association between HPV and human fertility through a systematic literature review.

## 1. Introduction

Human papillomavirus (HPV) is a common sexually transmitted virus. HPV is a DNA virus infecting the squamous epithelium of the reproductive system, the anal transformation zone, the mucosal epithelium of the larynx, the tonsils, and the oropharynx [1,2,3]. It is transmitted through sexual contact by all contact points and its incidence is relatively high in both genders regardless of the socioeconomic status and geographic location. HPV, according to the type, is responsible for the formation of benign lesions (warts or papillomas) and intraepithelial neoplasia, which in combination with specific and close-related risk factors, similar to those of cervical cancer, such as life style factors, multiple sexual partners, and infectious agents may lead to carcinogenesis [4,5]. HPV 16, 18, and 31 genotypes were the most prevalent high risk (HR) HPV types, while HPV 6 and 62 were the most prevalent low risk (LR) types in a group of 152 women who participated in a study investigating the relationship between HPV and cervical cancer in Egypt [6]. The HPV 52 and HPV 16, 58, 39, and 51 demonstrated the HP-HPV infections, while LR-HPV was HPV 81 followed by HPVs 6, 11, and 44 in a study investigating the correlation between HPV genotypes and cervical carcinoma [7]. HPV genotyping is of great importance in fertility/infertility diagnosis [8]. Recent studies propose a relationship between HPV and reduced fertility as well as a change in the outcome of the assisted reproductive technology [8,9]. The purpose of this work is to review the facts on HPV and its correlation with fertilization and it may be a useful tool for clinicians managing HPV in reproduction health care.

## 2. HPV and Semen

Several viral infections, such as human immunodeficiency virus or hepatitis B and C viruses may infect directly the male gametes, spermatozoa or indirectly the male reproductive track, causing indirectly male infertility [10,11,12,13,14,15]. Reduced fertility is reflected by altered sperm parameters, in increased sperm DNA fragmentation, and in increased sperm aneuploidy. It is thought that HPV is attached to the spermatozoa in two distinct sites along the equatorial region of the spermatozoon’s head, similarly to other viruses infecting the sperm. The presence of glycosaminoglycans or other soluble substances at the surface of the spermatozoa seems to mediate the interaction and binding of HPV on the spermatozoa. Recent data suggest that L1 capsid protein of the HPV and the glycosaminoglycan syndecan-1 are collocated at the equatorial segment of the spermatozoa head [16,17,18]. What is more, the equatorial segment is the site of the spermatozoon that binds and subsequently fuses with the plasma membrane of the oocyte [19]. Correlating all the above data we can assume that there is probably a negative impact of HPV in fertilization. Moreover, it has been demonstrated that impairment of sperm parameters maybe HPV genotype dependent. Specifically, in a study conducted by Connelly and colleagues (2001), it was found that HPV DNA 16 and 31 caused DNA breakages, proposing that these HPV genotypes may adversely impact on the developmental potential of embryos. Moreover, HPV DNA 18, 33 and 6/11 seemed not to affect the sperm parameters [20]. Additionally, it has been suggested by Lee and colleagues (2002) that exposure of sperm samples to HPV genotypes 6b/11, 16, 18, 31, and 33 preferentially degrade various exons of the involved genes, decreasing therefore the motility and the fertilizing potential of the spermatozoa [21]. In a clinical study conducted by Garolla and colleagues, it was found that the infection of spermatozoa with HPV was associated with the level of antisperm antibodies, suggesting indirectly that sperm samples positive to Mar tests should be considered for HPV infection [22]. In contrast to other viral infections, HPV does not affect the sperm chromosome number [14]. A large number of studies tend to be in line with the previously mentioned data, suggesting that HPV is related to bad sperm quality (reduced sperm motility, reduced total sperm count, reduced sperm count with normal morphology and reduced viability) [23,24,25]. On the other hand, a recent study failed to notice any relation between HPV presence in sperm and alteration of sperm parameters [26], while similar results were also found in different studies in males investigating their semen’s fertilization capability [27,28,29].

## 3. HPV and Early Stages of Embryogenesis

There is no doubt that spermatozoa can transfer viral DNA to the embryo through the fertilization process [30,31,32]. Studies in rodents have shown successful ovum fertilization from HPV infected spermatozoa [33]. Viral gene expression in the inner cell mass and trophectoderm was noted in the resulting blastocysts [34]. Studies using hamster egg fertilized by human spermatozoa (HEPT test) revealed that the gene expression of E6, E7, and L1 and the subsequent production of the proteins resulted in increased DNA fragmentation of the blastocysts and death of the trophoblasts [34]. In a study by Henneberg and colleagues investigating the relationship between HPV DNA exposure and embryo development, it was demonstrated that both genotypes HPV-16 and HPV-18 have embryo stage-specific effects [35]. Last, Gomez and colleagues found that the apoptosis rate was 3- to 6-fold greater in infected than in non-infected trophoblasts resulting in placental dysfunction and spontaneous preterm delivery [36].

## 4. HPV, Female Fertility and Spontaneous Abortions

In contrast to studies regarding male fertility and how it is affected by HPV, studies examining the impact of HPV on female fertility are limited. Most studies investigate the effect of the virus on the female reproductive system. A study from which we could acquire relative information is the one that investigates the relation between HPV infection and pregnancy outcome [8]. Some previous studies [36,37,38] have suggested a relation between virus infection and spontaneous abortions or premature rupture of membranes, a finding that was not confirmed by other studies [39,40]. Furthermore, a very recent study investigating 226 infertile couples for the impact of sperm HPV prevalence on spontaneous or assisted pregnancies, live births and miscarriages found a reduction in natural and assisted cumulative pregnancy rate and an increase in miscarriage rate regarding patients with HPV positive sperm cells [41]. Blastocysts that have been derived after insemination with HPV-infected spermatozoa may have lower implantation potential due to the infection of trophoblast cells of the blastocyst, implying indirectly the increased risk of abortion in these couples [42,43,44].

## 5. HPV and Assisted Reproductive Technology (ART)

It has been reported that HPV presence in sperm during assisted reproductive technology leads to lower rates of fertilization and increased rates of abortion. Researchers assume that during ART, HPV may intervene in the mechanisms of acrosome reaction, in the interplay between spermatozoa and ovum and in sperm-egg fusion. This is due to HPV binding on the spermatozoa head or tail segments, causing a reduction in acrosome functionality and capability [45].

The need for the development of a protocol to purify HPV infected sperm before its usage in ART has already been proposed by some researchers [46]. In serum-positive couples undergoing ART, a protocol involving enzymatic clearance has been used, which purifies sperm for a safe conception. A modified swim-up with added Heparinase III is used to remove HPV-DNA from infected semen samples [47].

### 5.1. Intrauterine Insemination

Studies have investigated the impact of HPV infection during IUI in infertile couples [41,48]. In a recent study by Depuydt and colleagues [48] found that HPV positive women undergoing IUI treatments have lower chances to achieve pregnancy. More specifically, among 590 women undergoing 1529 intrauterine insemination cycles, those suffering from an HPV infection had a six time less chance to become pregnant (1.87%) compared to women not infected by HPV (11.4%). It is worth mentioning from the above study that there was an 11% prevalence of the virus in each cycle [48]. In a more recent study by Garolla and colleagues, investigating 226 infertile couples, it was demonstrated that couples with infertility problems were allocated into two groups, depending on HPV presence in spermatozoa detected by fluorescence in situ hybridization (FISH). Among couples undergoing intrauterine insemination, a clinical pregnancy occurred in 12 out of 60 (20%) couples belonging to the non-infected group and in 2 out of 21 (9.5%) couples of the HPV-positive group [41].

### 5.2. In vitro Fertilization

It is known that women with infertility problems, with an indication for IVF treatment, exhibit twice more often HPV-related abnormal cervical cytology or high grade cervical lesions compared to the general population [5]. There are studies whose results have shown that there is no relation between HPV-16 and IVF outcomes [49] and no statistically significant differences in pregnancy rates between HPV-positive and healthy couples undergoing IVF/ET [50]. In one of the first studies evaluating the effect of HPV on IVF outcome by Spandorfer and colleagues [9], it was shown that HPV infection was detected in 16% of all patients. Pregnancy rate in women undergoing IVF treatment was two-fold higher compared to HPV-positive women (57% vs. 23.5%). It is worth mentioning that in that study [9] the number of oocytes retrieved as well as the embryo quality was not affected by the HPV infection. The spontaneous abortion rate did not differ significantly between the two groups [9]. In addition to the above-mentioned data, Garolla and colleagues [41] in a recent study allocated couples into two groups, depending on the presence of the virus in the sperm, and they underwent intracytoplasmic sperm injection (ICSI). Among the healthy couples, clinical pregnancy was achieved in 40 out of 98 (40.8%) of them, while among couples with HPV-positive sperm only 6 of 33 (18.2%) couples achieved clinical pregnancy, although fertilization rates following ICSI were similar in the two groups. It has been also shown that the proportion of blastocyst formation was significantly lower in the HPV infected group compared to the healthy group (27.3% vs. 54.1%, respectively). The HPV-negative group had a spontaneous pregnancy rate of 8.1%, in contrast to the HPV-positive couples, where the percent of spontaneous pregnancies was zero [41].

A recent novel meta-analysis performed by Siristatidis and colleagues [51] is the first one that complexes the HPV infection and ART outcome. Including all the relative publications concerning HPV and ART outcome, it was found that there is no enough evidence to draw solid conclusions regarding the impact of HPV infection in women on assisted reproductive outcomes. Further to the above founding, it was also reported that when HPV infection is present in the male partner, it appears that there is a negative effect on live birth/ongoing pregnancy rate and an increase in the miscarriage rate [51]. Lastly, following the previous novel meta-analysis, Xiong and colleagues [52], performed another meta-analysis relating the HPV risk with spontaneous abortion and spontaneous preterm birth (sPTB). It was found that HR-HPV infection was a risk for sPTB. Moreover, HPV infection was found to be independent of the clinical pregnancy rate and spontaneous abortion [52], results similar to those of Siristatidis and colleagues. These two meta-analyses revealed that there is no evidence to blame HPV for ART outcome, apart from the preterm delivery.

## 6. HPV and Cryopreservation

A significantly higher percentage of HPV-infected sperm cells compared to controls were found in a study that compared the percentage of presence of HPV in sperm cells from cryovials between oncology patients and healthy controls [53]. It was suggested in that study that it is not known whether the infected sperm are able to cross-contaminate cryovials and impair the outcome of assisted reproduction techniques, but indirectly implies that HPV can retain its DNA integrity in ultra-cooling conditions [53]. It is recommended that HPV examination should be performed in all semen samples before sperm banking, since HPV is very common worldwide and the actual incidence of infection has not been accurately calculated [53]. Moreover, it is suggested that HPV-positive sperm samples should be cryopreserved in different tanks from that of healthy samples, since cross-contamination is a possibility although it has been not yet proved.

## 7. Conclusions

HPV infection seems to be significantly related to negative effects on both the female and male reproductive system and consequently on the normal reproductive function. Based upon the existing studies, viral infection in men may lead to bad sperm quality (mainly asthenozoospermia) and increased rates of antisperm antibodies. On the other hand, more research is needed on the viral effect on female fertility. Although data are conflicting, it seems that spontaneous abortion rate and premature rupture of membranes during pregnancy are higher in cases of a coexisting HPV infection. The role of HPV in assisted reproductive technology is not clearly defined. We can certainly support that viral infection has no positive effects on IVF outcomes, as the existing studies suggest either a negative impact (reduced pregnancy rates) or no effect at all. More studies and in vitro research on human blastocysts are needed in order to evaluate the effect of HPV on early stages of embryonic development, because most of the currently available data are derived from research in mice.

HPV detection and genotype determination can be helpful, especially in cases of unexplained infertility in order to select the proper therapeutic protocol. For that reason, further research is required regarding the need for sperm clearance and sperm free-HPV before its usage in assisted reproductive technology. Nowadays, vaccination of both males and females is an urgent need. Immunization against the virus will act as the starting point to reduce the viral prevalence and subsequently its effects on the general health and fertility of both genders. It is certain that in the near future more data will emerge regarding the role of HPV in reproductive medicine and its effects on assisted reproduction. These data may change the way clinicians manage HPV infection in the context of assisted reproduction.
